# A biopsychosocial path model linking tobacco smoking to the Native American chronic pain disparity: findings from the Oklahoma Study of Native American Pain Risk

**DOI:** 10.1093/abm/kaag035

**Published:** 2026-06-15

**Authors:** Jamie L Rhudy, Michelle R vanDellen, Joseph W Ditre, Brandon W Jones, Taylor V Brown, Claudia N Vore, Kayla N Trevino, Aleiyah M Fields, Travis S Lowe, Joanna O Shadlow

**Affiliations:** TSET Health Promotion Research Center, The University of Oklahoma Health Sciences, Tulsa, OK 74135, United States; Department of Health Promotion Sciences, The University of Oklahoma Health Sciences, Tulsa, OK 74134, United States; Department of Psychology, The University of Tulsa, Tulsa, OK 74104, United States; TSET Health Promotion Research Center, The University of Oklahoma Health Sciences, Tulsa, OK 74135, United States; Department of Health Promotion Sciences, The University of Oklahoma Health Sciences, Tulsa, OK 74134, United States; Center for Health Behavior Research & Innovation, Syracuse University, Syracuse, NY 13244, United States; Department of Psychology, Syracuse University, Syracuse, NY 13244, United States; Department of Psychology, The University of Tulsa, Tulsa, OK 74104, United States; Department of Psychology, The University of Tulsa, Tulsa, OK 74104, United States; Department of Psychology, The University of Tulsa, Tulsa, OK 74104, United States; TSET Health Promotion Research Center, The University of Oklahoma Health Sciences, Tulsa, OK 74135, United States; Department of Psychology, The University of Tulsa, Tulsa, OK 74104, United States; Department of Anthropology and Sociology, The University of Tulsa, Tulsa, OK 74104, United States; Department of Psychology, Oklahoma State University, Tulsa, OK 74106, United States

**Keywords:** smoking, chronic pain, ethnic differences, Native Americans, interpersonal discrimination, stress

## Abstract

**Background:**

Among US groups, Native Americans (NAs) have the highest rates of smoking and chronic pain. No study has examined whether smoking contributes to NA chronic pain disparities.

**Purpose:**

We tested a biopsychosocial model linking smoking with chronic pain among NAs.

**Methods:**

Social, psychological, and biological variables associated with chronic pain risk and smoking were assessed in healthy, pain-free NAs and non-Hispanic Whites (NHWs). Participants were followed for 5 years to assess who did (*N* = 49) and did not (*N* = 151) develop chronic pain (pain rated ≥3/10 on most days lasting ≥3 months).

**Results:**

Native Americans had higher odds of smoking and developing chronic pain than NHWs, and smoking predicted chronic pain at 5 years (OR = 3.86, 95% CI, 1.59-9.35), even after controlling for age, sex, income, and education, but NA ethnicity did not confer greater chronic pain risk among those that smoke. A path analysis suggested that smoking contributed to the NA chronic pain disparity via 4 indirect paths. One linked NA ethnicity to chronic pain via smoking. Others suggested that the higher smoking rate in NAs was partially explained by interpersonal discrimination, and that cardiometabolic load (stress-related wear-and-tear on cardiovascular/metabolic systems) and impaired physiological pain inhibition (assessed by quantitative sensory testing) linked smoking to NA chronic pain.

**Conclusions:**

Smoking fits within a biopsychosocial model of NA chronic pain risk. Discrimination is linked to higher rates of smoking among NAs; smoking is associated with the NA chronic pain disparity; and higher cardiometabolic load and impaired pain inhibition link smoking to NA pain disparities.

## Introduction

Smoking combustible tobacco is implicated in the initiation and maintenance of chronic pain,^1^ including low back pain,^2^ rheumatoid arthritis,^3^ headaches,^4^^,^^5^ sciatica,^6^ and chronic persistent pain,^7^ and quitting smoking improves pain in chronic pain patients.^8^^,^^9^ This relationship is at least partly due to a disruption of pain regulation processes.^10–12^ Supporting this, lifetime smoking exposure (ie, pack-years) is associated with greater physiological pain amplification in chronic pain-free individuals.^10^ Similarly, chronic nicotine exposure in rodents induces reversible neuroplasticity within pain regulation circuitry that causes pain amplification.^13^

Native Americans (NAs) have the highest rates commercial tobacco smoking^14^ and chronic pain^15^ of all US racial/ethnic groups. To date though, no study has assessed whether smoking contributes to NA chronic pain risk and whether biopsychosocial mechanisms promote the smoking-pain relationship. Understanding these relationships is critical for informing prevention and intervention efforts. This is especially important as smoking rates among NAs have been rising,^14^ which may contribute to higher rates of chronic pain in this population.

The parent study from which these data were collected (ie, Oklahoma Study of Native American Pain Risk; OK-SNAP) was conducted to identify mechanisms that contribute to NA pain disparities. Biopsychosocial risk variables were assessed in healthy, pain-free NAs and non-Hispanic Whites (NHWs), and then they were followed to determine who developed chronic pain. We found NAs had 4 times the odds of developing chronic pain at the 5-year follow-up (OR = 4.03; NA_incidence_ = 28%, NHW_incidence_ = 12%),^16^ a disparity partly explained by psychosocial stressors (ie, discrimination), stress-reactions (ie, perceived stress, allostatic load = stress-related wear-and-tear on physiologic systems), enhanced pain amplification (ie, temporal summation of pain; TS-pain), and impaired pain inhibition (ie, conditioned pain modulation; CPM).^16^^,^^17^ Those findings led to a proposed model of NA chronic pain risk that posits that discrimination is an upstream promoter of stress and allostatic load, that subsequently results in increased pain amplification and impaired pain inhibition, that in turn increases risk for future chronic pain among NAs.^16^

**Figure 1:**
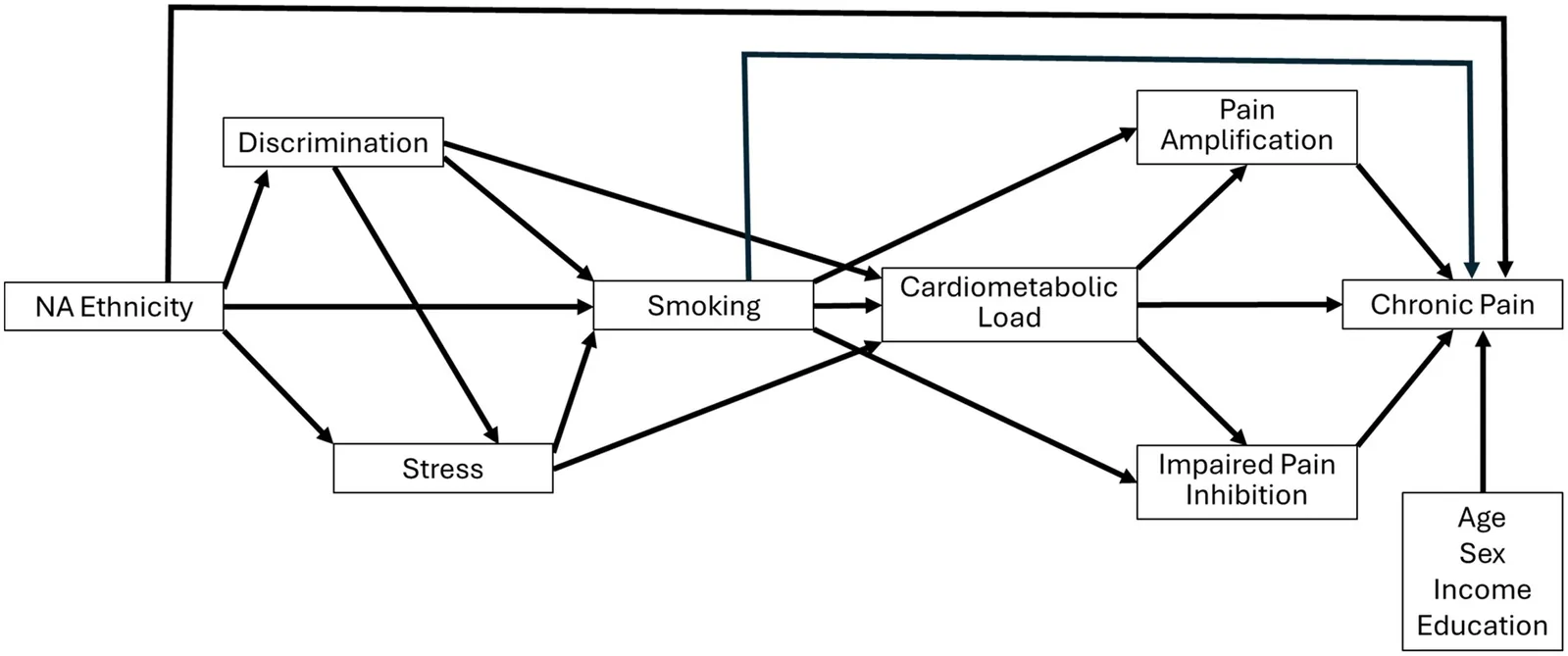
Proposed path analysis incorporating smoking into our current model of Native American (NA) chronic pain risk. We propose that smoking increases NA pain risk as a result of exposure to discrimination and stress, which will have downstream consequences by increasing cardiometabolic load, increasing pain amplification, and impairing pain inhibition. This model controls for age, sex assigned at birth, income, and education, which are variables known to increase pain risk. All variables were assessed during 2 laboratory visits, except chronic pain status which represents the presence or absence of chronic pain at the 5-year follow-up following enrollment.

Given the high rate of commercial tobacco smoking among NAs,^14^ smoking may also contribute to the NA chronic pain disparity through associations with variables within this previously proposed NA pain risk model.^1^ Indeed, factors associated with NA chronic pain risk are also associated with smoking among NAs.^16^^,^^17^ For example, discrimination, major life stressors, and stress are associated with NA smoking.^18–21^ Additionally, smoking is associated with higher allostatic load.^22–24^ Further, 1 cross-sectional study found that discrimination was associated with experiencing pain and pain-related interference which in turn was associated with a greater odds of smoking among Two-Spirit NAs.^18^ However, no study has linked smoking to other variables within the NA pain risk network and then used them to predict future chronic pain onset. We use the previously reviewed literature to situate smoking within the established NA pain risk model.^16^^,^^17^ Specifically, we predict that discrimination and psychological stress will be associated with greater risk of smoking, and that discrimination, stress, and smoking will increase allostatic load (measured by cardiovascular and metabolic markers in OK-SNAP). We predict that these factors will increase pain amplification (ie, increased TS-Pain) and impair pain inhibition (ie, reduced CPM) that in turn will contribute to the NA chronic pain disparity (Figure 1). We leveraged data from the parent study (chronic pain *n* = 49; pain-free *n* = 151) to test 3 aims: (1) establish whether a NA smoking disparity exists among parent study participants, (2) determine whether smoking at baseline is prospectively associated with chronic pain onset at 5-year follow-up, and (3) test a biopsychosocial model that situates smoking within the established NA chronic pain risk framework (Figure 1).

## Methods

This study represents a secondary analysis from OK-SNAP. A thorough description of participants, measures, and methods are described elsewhere.^16^ Study method and results are reported following the Strengthening the Reporting of Observational Studies in Epidemiology statement for cross-sectional studies.^25^ All procedures were approved by Institutional Review Boards of The University of Tulsa, University of Oklahoma Health Sciences, Cherokee Nation, and the Indian Health Service Oklahoma City Area Office. This manuscript was reviewed and approved by the Cherokee Nation and Oklahoma City Area Indian Health Service IRBs.

### Participants

Healthy, pain-free participants were enrolled so that they could be followed to prospectively determine chronic pain onset. Initial enrollment occurred enrolled between March 2014 and October 2018, whereas follow-up data were collected through October 2024. Targeted enrollment was based on a power analysis of the parent study^26^ and a flow chart of the participants at each study stage can be found here.^16^ NHWs (*n* = 114) endorsed White race and non-Hispanic ethnicity, whereas NAs (*n* = 86) endorsed NA/American Indian race and provided a Certificate of Degree of Indian Blood or tribal membership card. Tribal affiliations are not reported to respect tribal confidentiality. Exclusion criteria at enrollment were: (1) <18 years old; (2) self-reported history of cardiovascular, neuroendocrine, musculoskeletal, neurological disorders; (3) current self-reported chronic pain or acute pain problems that were assessed from a health status questionnaire and interview; (4) BMI ≥ 35 (due to difficulties recording the nociceptive flexion reflex, NFR)^27^; (5) current use of anti-depressant, anxiolytic, analgesic, stimulant, or anti-hypertensive medication; (6) self-reported psychotic symptoms or substance use problems; and/or (7) an inability to read and speak English.

### General procedures

Predictor variables were collected during 2 laboratory visits, whereas chronic pain status was assessed from surveys collected every 6 months for 60 months following enrollment. To limit the number of parameters/statistical tests in the present study, only those predictors previously associated with NA chronic pain risk were considered.^16^ At the first visit, participants were given a thorough overview of procedures before they provided verbal and written informed consent. On 1 visit, several physiological tasks were administered, including TS-pain and CPM, and 5- to 20-min breaks were provided between tasks during which questionnaires assessed psychosocial variables.^16^

### Assessing chronic pain onset

Every 6 months, participants were sent a survey with 19 body sites (head, neck, face, shoulder, upper back, lower back, arms, elbows, wrists, hands, buttocks, hips, chest, abdomen/pelvis, legs, knees, ankles, feet, other) and asked whether they had pain at each site that was not due to aches/pains that was fleeting or minor. If endorsed, participants estimated the date the pain began and rated the average pain on a 0-10 pain scale (0 = “no pain,” 10 = “pain as bad as could be”). “Other” pain was recoded into one of the other 18 sites (eg, back of thigh=leg pain). A participant was said to have chronic pain if they had pain rated ≥3, on more days than not, that lasted ≥3 months and began after study enrollment. The most frequently reported chronic pain was lower back (47%), knee (27%), shoulder (16%), and foot (16%).^16^ The primary dependent variable was a dichotomous variable coding 0 = no chronic pain *at any body site* at any follow-up or 1 = chronic pain *at any body* site.

### Questionnaires

#### Demographics

A demographics questionnaire assessed sex assigned at birth, age at enrollment, education level, and annual household income.

#### Smoking status

Three questions assessed tobacco use. The first asked “Do you use tobacco?” A second question assessed amount of use. A third item assessed length of use (in years) was used for descriptive purposes. A question asking about the specific tobacco type was not administered, rather tobacco type was inferred from the question assessing amount. Given that all reported tobacco use in packs/day, it was assumed that all persons endorsing tobacco use smoked cigarettes. So a dichotomous variable was created to code for smoking (0 = person that does not smoke, 1 = person that smokes).

#### Discrimination

The 9-item Everyday Discrimination Scale^28^^,^^29^ assessed interpersonal discrimination. Items were reverse scored and averaged (range 1-6) so that higher scores indicated greater discrimination (Cronbach’s alpha for NA α = .897; NHW α = .917).

#### Stress

A principal components analysis was used to create a composite stress/distress score from the 14-item Perceived Stress Scale (PSS)^30^ and the Global Severity Index (GSI) of the Symptom Checklist-90-Revised.^31^ Cronbach’s alpha was high for both scales (PSS: NA α = .858; NHW α = .861; NA α = .975; NHW α = .966). The component accounted for 85.6% of the variance in the 2 variables, with higher component scores indicating higher stress/distress.

### Cardiometabolic load

High body mass index (BMI), high blood pressure (BP), and low root mean square of successive differences (RMSSD, a measure of heart rate variability) are markers of stress-related wear-and-tear on cardiovascular and metabolic systems.^32^ We created a single cardiometabolic load component from these variables using principal components analysis (PCA), as we have done previously.^16^^,^^17^ BMI was assessed from height and weight. Resting systolic and diastolic BP (Dinamap; Tampa, FL) was assessed 3 times at Visit 1 (3-minute inter-test interval) and averaged. RMSSD was assessed from 2-lead electrocardiogram sampled at 1000 Hz (Grass Technologies, West Warwick, RI; Model 15LT and AC Module 15A54) during two 5-minute resting periods that were analyzed using HRV software.^33^ RMSSD from the 2 periods was averaged. The cardiometabolic load component accounted for 50% of the variance in the 4 variables, with higher component scores indicating greater load.

### Assessment of pain regulation

#### Pain inhibition: CPM

Conditioned pain modulation assesses pain inhibition by assessing pain ratings and the NFR (marker of pain signaling in the spinal cord^27^) in response to painful electric stimuli before and during hand submersion in painfully cold circulating water (10 ± 0.1 °C). In healthy participants, hand submersion engages physiological processes that inhibit pain and NFRs from the electric stimuli. We have shown that NFR inhibition during CPM (ie, CPM-NFR; physiological marker of spinal pain neuron inhibition), but not pain inhibition during CPM (ie, CPM-pain; marker of pain experience inhibition), is associated with chronic pain risk.^16^^,^^17^ Thus, we focused only on CPM-NFR.

The intensity of the painful electric stimulations was calibrated to be above each participant’s pain threshold and NFR threshold (ie, stimulus intensity that elicits the NFR).^16^ NFR magnitudes, assessed from electromyogram of the hamstring muscle responsible for the reflex (Grass Technologies, West Warwick, RI; Model 15LT and AC Module 15A54), in response to electric test stimuli were used to assess NFR inhibition.^34^ CPM-NFR was defined as the average of the NFRs during the cold water minus the average of the NFRs during the baseline phase. Lower scores mean more NFR inhibition and higher scores mean less inhibition.

#### Pain amplification: TS-pain

Temporal summation of pain assesses individual differences in pain amplification.^35^ It refers to the increase (summation) in pain experience that occurs in response to a series of painful stimuli applied to the same site. In healthy participants, the last stimulus hurts more than the first even though the intensity of the stimulus does not change. TS-pain was assessed by delivering 5 series of 3 painful electric stimulations and 5 single painful electric stimulations. The electric stimulation intensity was calibrated to the participant, similar to how it was for CPM.^16^ After each stimulus, participants rated their pain using a visual analog scale ranging from 0 = no pain sensation to 100 = most intense pain sensation imaginable. TS-pain was defined as the difference in the average pain ratings in response to the last rating of the 3-stimulus series minus the average pain ratings of the single painful electric ­stimulations.^16^ Higher scores mean greater pain amplification.

### Data analyses

SPSS (IBM; v29) was used for data screening, independent samples *t*-tests, chi-squared analyses, and logistic regression analyses. Mplus (v8.11; Los Angeles, CA: Muthén & Muthén) using Bayesian estimation was used for the path analysis. For all analyses, ethnicity was coded 0 = NHW versus 1 = NA and smoking status was coded 0 = person who does not smoke versus 1 = person who smokes. Significance was set to α = .05 (2-tailed).

Skewed variables were transformed and outliers were identified using Wilcox’s MAD-median procedure (using recommended 2.24 cutoff).^36^ Outliers were winsorized using the next nearest non-outlier value (winsorized variables: systolic BP, diastolic BP, discrimination, perceived stress, psychological distress, CPM-NFR, and TS-pain). Transformed/winsorized variables were used in inferential statistics and effect size calculations (non-transformed means/SDs are reported in Table S1 for age, RMSSD, and GSI to aid interpretation). Ordinal variables (education, income) were treated as continuous variables in logistic regression and path analyses.

Logistic regression analyses were used to examine the impact of ethnicity on smoking status (aim 1) and smoking status on chronic pain status (aim 2), after controlling for age, biological sex, education, and income.^37^^,^^38^

The path analysis was based on evidence linking psychosocial variables (eg, discrimination, perceived stress/distress) to tobacco smoking,^18–21^ and evidence linking smoking to greater allostatic load,^22–24^ disrupted pain regulation,^10^^,^^13^ and chronic pain risk.^2–6^ Age, biological sex, education, and income were controlled.^37^^,^^38^ Statistical significance of path estimates was assessed using Bayesian 95% credible intervals. Bayesian models used Mplus default priors. Specifically, weakly informative Normal(0, 5) priors were used for all thresholds and those paths with smoking or chronic pain as outcomes, diffuse Normal priors (Normal[0, ∞]) were used for all intercepts and those paths that do not have smoking or chronic pain as outcomes, and inverse‑gamma IG(−1, 0) priors were used for all variances. Missingness on endogenous variables was handled in Mplus using Markov chain Monte Carlo estimation and multiple imputation. Missing values on exogenous variables (ie, income, education) were imputed prior to the path analysis using the expectation maximization algorithm in LISREL v8.8 (Scientific Software International; Lincolnwood, IL). All possible indirect/mediation effects linking ethnicity to chronic pain were evaluated using Mplus’ MODEL INDIRECT feature and 95% CIs.

## Results

### Participant characteristics

The sample was 53.5% female, 73.5% single, and 67% employed part- or full-time. Mean age was 28.33 (SD = 12.70). As noted elsewhere,^16^ NAs had higher BP, experienced more discrimination, stress, and distress, had higher cardiometabolic load and stress composite variables, and were more likely to develop chronic pain (Tables S1 and S2 present ethnic differences).

### Aim 1: Ethnic differences in smoking

There was an ethnic difference in smoking: 23.3% of NAs currently smoked, whereas 10.5% of NHWs currently smoked (χ^2^ = 5.910, *P* = .015). According to the logistic regression model, the adjusted odds ratio linking ethnicity to smoking was 2.949 (95% CI, 1.297-6.708) and the model explained 14% of the variance (Nagelkerke *R*^2^ = 0.138), indicating that NAs had 2.95 times the odds of smoking than NHWs, even after controlling for sex, age, and socioeconomic status. Among those that smoked, groups did not differ in average packs/day (NA = 0.704, SD = 0.572 vs NHW = 0.415, SD = 0.321; *P* = .121) or years of tobacco use (NA = 12.784, SD = 10.089 vs NHW = 13.664, SD = 12.901; *P* = .835).

### Aim 2: The smoking-chronic pain relationship

The adjusted odds ratio linking smoking status to chronic pain was 3.855 (95% CI, 1.589-9.351), indicating those who smoke had a 3.86 higher odds of developing chronic pain than those that do not smoke, even after controlling for sex, age, and socioeconomic status. The model explained 24% of the variance in chronic pain status (Nagelkerke *R*^2^ = .242). Despite the higher rate of chronic pain among people who smoke, the rate of chronic pain among people that smoke was similar in both ethnic groups; 50% of NHWs who smoke and 55% of NAs who smoke developed chronic pain (χ^2^ = 0.075, *P* = .784).

### Aim 3: A biopsychosocial model of smoking and chronic pain risk

**Figure 2:**
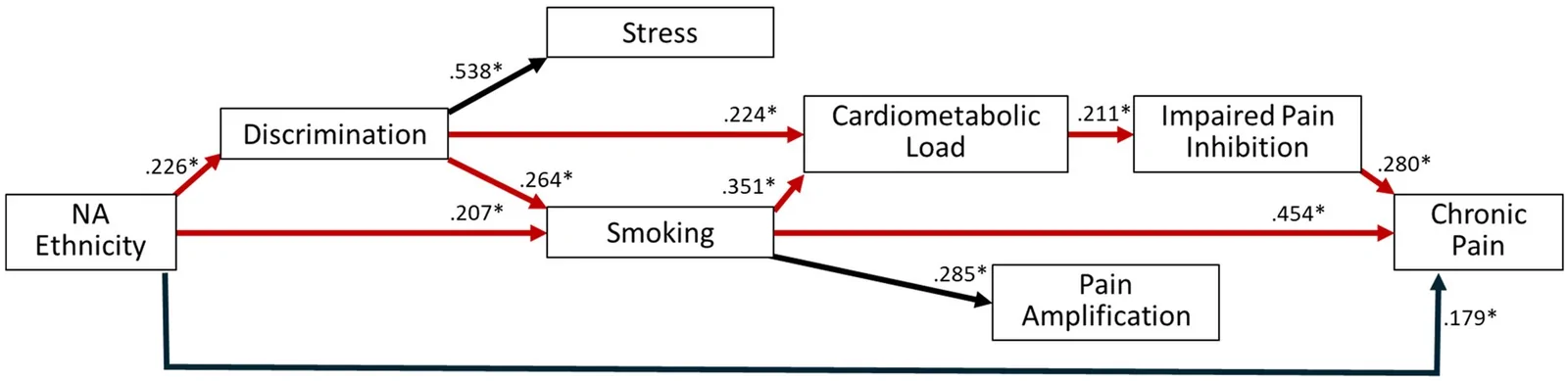
Results of the path analysis linking Native American (NA) ethnicity to chronic pain onset. Standardized coefficients are reported. *Significant according to 95% credible interval (only paths that are significant are depicted in the figure). Red arrows represent paths that contribute to the 5 significant indirect (mediated) paths linking NA ethnicity to chronic pain onset: (1) NA Ethnicity → Smoking → Chronic Pain; (2) NA Ethnicity → Discrimination → Smoking → Chronic Pain; (3) NA Ethnicity → Smoking → Cardiometabolic Load → Impaired Pain Inhibition (CPM-NFR) → Chronic Pain; (4) NA Ethnicity → Discrimination → Cardiometabolic Load → Impaired Pain Inhibition (CPM-NFR) → Chronic Pain; (5) NA Ethnicity → Discrimination → Smoking → Cardiometabolic Load→Impaired Pain Inhibition (CPM-NFR) → Chronic Pain. No control variable was associated with chronic pain after including the other predictors in the path model.

The path analysis found that being NA was associated with greater experiences of discrimination and higher odds of smoking (Table 1, Figure 2). Discrimination was associated with higher stress, higher cardiometabolic load, and higher odds of smoking. Smoking was associated with higher cardiometabolic load, higher pain amplification, and higher odds of developing chronic pain. Cardiometabolic load was associated with impaired pain inhibition (CPM-NFR) and impaired pain inhibition was associated with higher odds of developing chronic pain. After controlling for mediators and control variables, there was still a significant direct effect between ethnicity and chronic pain, suggesting that the mediation paths do not completely explain the NA chronic pain disparity.

**Table 1:** Standardized estimates and 95% credible intervals for relationships in the path analysis linking Native American ethnicity to chronic pain onset.^a^

	Standardized	95% CI	
**Predicting chronic pain status**	Estimate	Lower	Upper
** NA ethnicity**	**0.179**	**0.007**	**0.354**
** Smoke**	**0.454**	**0.189**	**0.690**
** Pain amplification (TS-pain)**	0.144	−0.073	0.340
** Pain inhibition (CPM-NFR)**	**0.280**	**0.093**	**0.453**
** Cardiometabolic load**	−0.027	−0.254	0.184
** Sex**	0.151	−0.027	0.313
** Age**	0.033	−0.092	0.176
** Education**	−0.006	−0.156	0.146
** Income**	−0.087	−0.252	0.078
**Predicting pain amplification (TS-pain)**	Estimate	Lower	Upper
** Smoke**	**0.285**	**0.045**	**0.502**
** Cardiometabolic load**	0.038	−0.151	0.222
**Predicting impaired pain inhibition (CPM-NFR)**	Estimate	Lower	Upper
** Smoke**	0.067	−0.175	0.294
** Cardiometabolic load**	**0.211**	**0.037**	**0.379**
**Predicting cardiometabolic load**	Estimate	Lower	Upper
** Discrimination**	**0.224**	**0.033**	**0.399**
** Smoke**	**0.351**	**0.146**	**0.523**
** Stress component**	−0.103	−0.284	0.077
**Predicting smoke**	Estimate	Lower	Upper
** Discrimination**	**0.264**	**0.063**	**0.450**
** Stress component**	−0.099	−0.322	0.125
** NA ethnicity**	**0.207**	**0.028**	**0.385**
**Predicting stress component**	Estimate	Lower	Upper
** Discrimination**	**0.538**	**0.427**	**0.632**
** NA ethnicity**	0.056	−0.056	0.175
**Predicting discrimination**	Estimate	Lower	Upper
** NA ethnicity**	**0.226**	**0.088**	**0.349**

Abbreviations: NA ethnicity, Native American (0 = non-Hispanic White, 1 = Native American); Smoke, smoking status (0 = people who do not smoke, 1 = people who smoke); TS, temporal summation; CPM-NFR, conditioned pain modulation of the nociceptive flexion reflex, a physiological marker of pain inhibition (more positive scores indicate impaired pain inhibition); Sex, sex assigned at birth (0 = male, 1 = female); Cardiometabolic load, component score generated from BMI, blood pressure, and heart rate variability; Stress component, component generated from perceived stress and psychological distress.

a Bolded standardized estimates are significant at *P* < .05 according to the 95% credible interval.

There were 5 significant indirect effects linking NA ethnicity to chronic pain (Table 2). The first only included smoking as a mediator. The second included discrimination and smoking. The third included smoking, cardiometabolic load, and impaired pain inhibition. The fourth included discrimination, cardiometabolic load, and impaired pain inhibition. The last included discrimination, smoking, cardiometabolic load, and impaired pain inhibition. Stress and pain amplification did not contribute to any indirect effects, although they were significantly related to other variables. None of the control variables was related to chronic pain when other predictors were included. The model explained 54% of the variance in chronic pain status (Table 3).

**Table 2:** Total, direct, and indirect effects and their 95% credibility intervals linking Native American ethnicity to chronic pain.^a^

	Standardized	95% CI	
Paths	Estimate	Lower	Upper
Total effect: NA ethnicity → CP	0.932	0.432	1.485
**Total indirect effect**	0.368	0.088	0.874
Direct effect: NA ethnicity → CP	0.531	0.022	1.045
**Indirect effect paths**			
** NA ethnicity → Smoking → CP**	0.260	0.027	0.700
** NA ethnicity → Discrimination → Smoking → CP**	0.073	0.011	0.220
** NA ethnicity → Smoking → Load → CPM-NFR → CP**	0.010	<0.001	0.044
** NA ethnicity → Discrimination → Load → CPM-NFR → CP**	0.007	<0.001	0.030
** NA ethnicity → Discrimination → Smoking → Load → CPM-NFR → CP**	0.003	<0.001	0.012

Abbreviations: NA ethnicity, Native American ethnicity (0 = non-Hispanic White, 1 = Native American); CPM-NFR, conditioned pain modulation of the nociceptive flexion reflex, a physiological marker of pain inhibition; Smoke, smoking status (0 = people who do not smoke, 1 = people who smoke); Load, cardiometabolic load component score; CP, chronic pain status (0 = no chronic pain, 1 = chronic pain).

a Estimates for which 0 is not contained in the credible interval are significant at *P* < .05.

**Table 3:** Variance explained (*R*^2^) and 95% confidence intervals in each dependent variable in the path analysis.

		95% CI	
Dependent variable	*R* ^2^	Lower	Upper
**Discrimination**	0.051	0.008	0.122
**Smoking status (0 = people who do not smoke, 1 = people who smoke)**	0.132	0.037	0.254
**Stress component score**	0.309	0.205	0.416
**Cardiometabolic load component score**	0.202	0.078	0.353
**Pain amplification (TS-pain)**	0.099	0.014	0.242
**Impaired pain inhibition (CPM-NFR)**	0.072	0.016	0.172
**Chronic pain (0 = no pain, 1 = chronic pain)**	0.538	0.353	0.722

Abbreviations: CI, confidence intervals (frequentist), because Bayes estimation does not provide credible intervals for *R*^2^ values; TS-pain, temporal summation of pain, a psychophysical measure of pain amplification; CPM-NFR, conditioned pain modulation of the nociceptive flexion reflex, a physiological measure of pain inhibition.

## Discussion

This study is the first to show that tobacco smoking contributes to future NA chronic pain onset thorough several biopsychosocial paths. As predicted, NAs had a higher odds of smoking than NHWs (23% vs 10.5%; OR = 2.95), even after controlling for age, sex, and socioeconomic status. The higher smoking rate among NAs was partly explained by discrimination, an effect that has been previously observed among Two-Spirits^18^ and NA adolescents.^19^ Thus, psychosocial stressors may increase the odds of smoking and pain among NAs. This is consistent with the Minority Stress Model that stipulates minoritized individuals are more likely to experience chronic, identity-related stressors that produce psychological and physiological burden and elevate risk for problematic coping behaviors.^39^ Our findings suggest that smoking serves as a method of coping with interpersonal discrimination, an identity-related stressor, that increases risk for cardiometabolic problems and chronic pain. Thus, tobacco smoking appears to be 1 pathway by which minority stress gets embodied: specifically, by increasing allostatic load, dysregulating pain mechanisms, and increasing chronic pain risk.^40^^,^^41^

Although discrimination was associated with higher psychological stress, stress was not associated with smoking. This contrasts with other non-NA studies. For example, a cross-national study found that higher stress was associated with a higher likelihood of smoking^42^ and a diary study found that daily fluctuations in stress maintained smoking behaviors.^43^ To rule out that the lack of association between stress and smoking was an artifact of our stress composite score, we conducted exploratory zero-order correlations between smoking and the PSS and GSI (not presented); neither correlation was significant (*P*s > .50). Thus, future research is needed to determine if cultural, contextual, or measurement issues contributed to this null finding.

Smoking was strongly related to chronic pain onset (adjusted OR = 3.86). However, the proportion of those who smoke that developed chronic pain was similar among NHWs and NAs (50% vs 55%), indicating that it is not a stronger relationship between smoking and pain that contributes to the NA pain disparity, but rather the higher smoking prevalence among NAs. This is supported by the 4 indirect paths involving smoking that mediated the relationship between NA ethnicity and chronic pain status. Two paths involved a direct connection between smoking and chronic pain: (1) Ethnicity → Smoking → Chronic Pain and (2) Ethnicity → Discrimination → Smoking → Chronic Pain. Prior studies with non-NA samples have shown that persons that smoke are more likely to develop various chronic pain conditions than persons that do not smoke^2–7^, but ours is the first to show that smoking contributes prospectively to the NA pain disparity. Moreover, discrimination, a psychosocial stressor, appears to elevate chronic pain risk by increasing the probability of smoking. Given established reciprocal relationships between smoking and pain,^1^ discrimination may initiate or amplify a smoking-pain cycle that contributes to higher rates of smoking^14^ and chronic pain^15^^,^^44^ among NAs.

The other 2 indirect paths included cardiometabolic load and impaired pain inhibition assessed at baseline: (1) Ethnicity → Smoking → Cardiometabolic Load → Impaired Pain Inhibition → Chronic Pain and (2) Ethnicity → Discrimination → Smoking → Cardiometabolic Load → Impaired Pain Inhibition → Chronic Pain. Allostatic load refers to wear-and-tear on cardiovascular, neuroendocrine, metabolic, and immune systems as a result of stress.^24^ OK-SNAP only assessed markers of cardiovascular and metabolic function due to budget constraints, so our variable focused on cardiometabolic load. Nonetheless, findings are consistent with studies that comprehensively assessed allostatic load in non-NA samples. For example, a national study of middle-aged women found that smoking was associated with higher allostatic load that in turn predicted a higher risk of cancer.^24^ Another national study of persons in midlife found that living in a lower income neighborhood (a social stressor) was associated with higher allostatic load, an effect that was partially mediated by smoking.^23^ And finally, a longitudinal study of 9-year-old children found that childhood stressors were associated with higher allostatic load in adolescence, an effect that was mediated by smoking.^22^ These last 2 studies are particularly relevant to our findings as they highlight that the effect of a stressor on physiological wear-and-tear is partly explained by smoking, a modifiable risk behavior. Our study extends this to NAs.

Our findings also suggest smoking impairs pain inhibition at baseline secondary to increased cardiometabolic load. This is consistent with studies showing that smoking is associated with impaired stress-induced analgesia in women^11^ and impaired endogenous opioids (neuropeptides responsible for pain ­inhibition).^12^ We also found that smoking increased pain amplification at baseline, but that path did not contribute to increased risk for chronic pain. To our knowledge, only 1 other study has demonstrated an effect of smoking on pain amplification.^10^ They measured pain amplification in response to capsaicin, the spicy ingredient in hot chili peppers, which is used to model inflammatory pain.^45^ We assessed pain amplification using TS-pain. Like capsaicin, TS-pain assesses pain amplification associated with spinal pain neuron hyperexcitability. Thus, smoking may promote central sensitization by exciting spinal neurons which amplify ascending nociception. However, our model suggests that this effect is less important for chronic pain risk than the impaired pain inhibition related to smoking and cardiometabolic load. But future research is needed to replicate these findings.

A fifth significant indirect path was observed, but it replicated a finding previously reported^16^ (Ethnicity → Discrimination → Cardiometabolic Load → Impaired Pain Inhibition → Chronic Pain), so it will not be discussed. Summed across all direct and indirect effects, almost 54% of the variance in chronic pain status was explained. Notably, despite the 5 significant indirect paths, the direct effect between NA ethnicity and chronic pain remained statistically significant, suggesting that there are mediators/mechanisms yet to identify.

## Clinical implications and future research

Given the strong association between smoking and chronic pain, and the disproportionate burden of both conditions among NAs, early identification of at-risk NAs may help guide preventive and intervention efforts. NAs that smoke non-ceremonial tobacco should be offered smoking cessation treatment, not only to prevent cardiovascular disease, cancer, and tobacco-related mortality, but also as a strategy to prevent/reduce chronic pain. Ideally, smoking cessation treatments should be culturally tailored,^46^ with a specific focus on non-ceremonial use, because ceremonial use does not contribute to tobacco-related health problems.^47^ Moreover, tailoring increases treatment engagement, acceptance, credibility, and efficacy.^48^

Native Americans seeking treatment for chronic pain should be screened for non-ceremonial tobacco smoking and offered an integrated approach that focuses on smoking, pain, and their inter-relationships. Currently, there are no well-established, empirically supported treatments that integrate these targets,^1^ highlighting a critical gap that future research should address. Because experiencing discrimination partly explained the higher rate of smoking and chronic pain in NAs, this also highlights the need for trauma-informed care.

Our findings also suggest that behavioral interventions that target cardiometabolic load, such as diet, physical activity, weight loss, as well as stress-reduction (eg, mindfulness, cognitive-behavioral therapy), may also reduce chronic pain risk. Interestingly, our findings suggest that targeting the physiological consequences of stress (ie, cardiometabolic load) may be more important than targeting the psychological experience of stress. Previously, we found that getting better sleep buffers against the harmful effects of discrimination on cardiometabolic load in NAs,^49^ thus treatments that target poor sleep may also help (eg, cognitive-behavioral therapy for insomnia). Some interventions that reduce cardiometabolic load (ie, exercise) also have a positive impact on pain inhibition,^50^ which is consistent with our model (Figure 2). In some cases, addressing cardiometabolic risks pharmacologically may be necessary (eg, statins, GLP-1 receptor agonists).

And finally, NA-specific resilience can combat many chronic pain risk factors. For example, cultural connectedness/continuity is cultural resilience and includes domains like NA spirituality, respect for culture/elders, community connection, holism, participation in indigenous traditions, traditional healing, and connection to land/home.^51^ Cultural connectedness can be increased by engaging in traditional ceremonies, language classes, and art workshops; receiving guidance from NA elders; and incorporating cultural practices into treatment.^52^ NAs that report more cultural connectedness have better health^53^ and experience less allostatic load in response to discrimination.^54^ Recently, we found that cultural connectedness was associated with higher self-reported pain resilience and better pain inhibition.^55^ Thus, cultural connectedness may help combat the smoking-pain relationship.

## Strengths and limitations

This study tested a novel hypothesis that smoking contributes to NA chronic pain risk. Other strengths include the prospective assessment of chronic pain onset, inclusion of biopsychosocial risk factors that influence NA smoking and pain, and use of advanced statistical analysis. Nonetheless, several limitations should be noted.

We did not biochemically verify smoking status. Although it is unlikely that participants reported smoking when they do not, it is possible that some participants that smoked did not accurately identify themselves. We also did not assess the use of other nicotine products (eg, e-cigarettes, vape pens) or ceremonial tobacco use; therefore, we could not fully account for the effects of nicotine (vs smoking) in our models. As we reported elsewhere,^16^ not all OK-SNAP participants completed follow-ups. Those who did not were more likely to be NA, had a lower education, had higher NFR thresholds. These differences may contribute to underestimation of the NA pain disparity, since these are risk factors for NA chronic pain.^16^ Further, generalizability of the findings may be impacted by our strict exclusion criteria (eg, high BMI, no centrally acting or pain medications, no current substance dependence).

Although we assessed chronic pain onset prospectively, all predictors were collected cross-sectionally 5 years prior. Thus, the directionality of the relationships among the predictors cannot be determined even though the proposed directionalities were based on theoretically and empirically established relationships in the literature. Further, we may have missed other important predictors that contribute to smoking-pain relationships (eg, inflammation, health insurance coverage, health care access) and we did not assess medication use in follow-up surveys, which could have affected pain and the classification of chronic pain at follow-up. Similarly, smoking status was not assessed at follow-up, so it is unknown whether smoking onset or cessation that occurred within the follow-up period contributes to chronic pain rates. Due to the relatively low base rate of chronic pain in our sample, we were also underpowered to look at important moderators of paths in the model, such as biological sex or pain type (eg, centralized vs local, neuropathic vs nociplastic vs inflammatory).

Native Americans in OK-SNAP represent tribes primarily from the Southern Plains; therefore, results may not generalize to other NAs. Moreover, there may be important within- and between-tribe differences that we did not assess that could have impacted relationships (eg, ceremonial tobacco use, traditional healing, cultural connectedness). Most participants reported lower back pain or other types of musculoskeletal pain, so our results may not generalize to other types of pain (eg, rheumatoid arthritis). We also had strict inclusion/exclusion criteria to control for potential confounds, but this may have limited generalizability to the broader NA population who experience other health inequities.

We restricted the predictors to those previously implicated in NA chronic pain risk to reduce the number of parameters and control familywise error rate. However, no other efforts to control Type I error were undertaken to maximize observing potentially important relationships in this novel dataset. Additionally, the complexity of the path model could have resulted in overfitting. For these reasons, results should be interpreted with caution until replicated. And finally, the relatively small sample of individuals that developed chronic pain may limit the precision of effect size estimates and the generalizability of the findings. Results should be replicated in larger samples.

## Summary

This study found that NAs were more likely to smoke than NHWs, an effect that was partly explained by greater experiences of discrimination. Importantly, this smoking disparity was associated with higher cardiometabolic load, impaired pain inhibition, and future chronic pain onset. This study represents the first empirical evidence that smoking contributes to the NA chronic pain disparity, identifies biopsychosocial factors that influence smoking-pain relationships, and provides insights into treatment targets to eliminate NA inequities in smoking and chronic pain.

## Supplementary Material

kaag035_Supplementary_Data

## Data Availability

De-identified data from this study are not available in a public archive. De-identified data from this study will be made available (as allowable according to institutional IRB standards) by emailing the corresponding author.

## Transparency statements

**Study registration**. This study was not formally registered.

**Analytic plan pre-registration**. The analysis plan was not formally pre-registered.

**Analytic code availability.** Analytic code used to conduct the analyses presented in this study are not available in a public archive. They may be available by emailing the corresponding author.

**Materials availability.** Materials used to conduct the study are not in a public archive, but those available can be obtained by emailing the corresponding author.
