# Supplementary material for: A biopsychosocial path model linking tobacco smoking to the Native American chronic pain disparity: findings from the Oklahoma Study of Native American Pain Risk
Source: Ann Behav Med. 2026 Jun 15;60(1):kaag035. doi: 10.1093/abm/kaag035 (PMC13265374; doi:10.1093/abm/kaag035)
Supplement: kaag035_Supplementary_Data [file kaag035_supplementary_data.zip › Supplemental Table 2 - Group Diffs Catagorical Vars.docx]

|  |  |  |  |  |  |  |  |  |  |  |
| --- | --- | --- | --- | --- | --- | --- | --- | --- | --- | --- |
|  | NHW | (N=114) |  | NA | (N=86) |  |  |  |  |  |
|  | N | % |  | N | % |  | χ^2^ | p |  | Phi |
| **Female Sex** | 57 | 50.0% |  | 50 | 58.1% |  | 1.305 | .253 |  | 0.081 |
| **Marital Status** |  |  |  |  |  |  | 0.619 | .734 |  | 0.056 |
| Single | 87 | 76.3% |  | 60 | 71.4% |  |  |  |  |  |
| Married/Cohabitating | 21 | 18.4% |  | 19 | 22.6% |  |  |  |  |  |
| Separated/Divorced/Widowed | 6 | 5.3% |  | 5 | 6.0% |  |  |  |  |  |
| **Employment Status** |  |  |  |  |  |  |  |  |  |  |
| ≥40 hrs/week | 26 | 23.2% |  | 24 | 27.9% |  | 6.028 | .110 |  | 0.174 |
| <40 hrs/week | 48 | 42.9% |  | 35 | 40.7% |  |  |  |  |  |
| Student/Retired | 16 | 14.3% |  | 4 | 4.7% |  |  |  |  |  |
| Unemployed | 22 | 19.6% |  | 23 | 26.7% |  |  |  |  |  |
| **Education Level** |  |  |  |  |  |  |  |  |  |  |
| <High School / High School | 19 | 16.7% |  | 20 | 23.3% |  | 1.449 | .485 |  | 0.085 |
| Partial College | 57 | 50.0% |  | 38 | 44.2% |  |  |  |  |  |
| College / Prof or Grad School | 38 | 33.3% |  | 28 | 32.6% |  |  |  |  |  |
| **Income Level** |  |  |  |  |  |  |  |  |  |  |
| <$9999 | 44 | 38.6% |  | 24 | 27.9% |  | 3.244 | .662 |  | 0.127 |
| $10K-14999 | 13 | 11.4% |  | 14 | 16.3% |  |  |  |  |  |
| $15K-24999 | 15 | 13.2% |  | 15 | 17.4% |  |  |  |  |  |
| $25-34999 | 9 | 7.9% |  | 8 | 9.3% |  |  |  |  |  |
| $35K-49999 | 10 | 8.8% |  | 8 | 9.3% |  |  |  |  |  |
| >$50K | 23 | 20.2% |  | 17 | 19.8% |  |  |  |  |  |
| **Current Chronic Pain** | 16 | 14.0% |  | 33 | 38.4% |  | 15.696 | <.001 |  | 0.280 |
| **Current Smoker** | 12 | 10.5% |  | 20 | 23.3% |  | 5.910 | .015 |  | 0.172 |

Supplemental Table 2. Ethnic Group Differences in Categorical and Ordinal Variables
