# Supplementary material for: A biopsychosocial path model linking tobacco smoking to the Native American chronic pain disparity: findings from the Oklahoma Study of Native American Pain Risk
Source: Ann Behav Med. 2026 Jun 15;60(1):kaag035. doi: 10.1093/abm/kaag035 (PMC13265374; doi:10.1093/abm/kaag035)
Supplement: kaag035_Supplementary_Data [file kaag035_supplementary_data.zip › Supplemental Table 1 - Group Diffs Continuous Vars.docx]

Supplemental Table 1. Ethnic group differences in continuous-like variables

|  |  |  |  |  |  |  |  |  |  |  |  |  |  |  |
| --- | --- | --- | --- | --- | --- | --- | --- | --- | --- | --- | --- | --- | --- | --- |
|  |  |  | NHW (N=114) |  |  |  | NA (N=86) |  |  |  |  |  |  | 95% CI for d |
|  | N | M | SD |  | N | M | SD |  | t-test | p-value |  | Cohen's d | Lower | Upper |
| Age (years) | 114 | 28.149 | 13.401 |  | 86 | 28.558 | 11.792 |  | -0.570 | 0.569 |  | -0.081 | -0.361 | 0.199 |
| BMI (kg/m^2^) | 113 | 24.133 | 3.791 |  | 85 | 25.259 | 4.399 |  | -1.932 | 0.055 |  | -0.277 | -0.560 | 0.006 |
| Resting Systolic BP (mmHg) | 113 | 114.201 | 11.520 |  | 84 | 118.722 | 12.718 |  | -2.606 | 0.010 |  | -0.375 | -0.660 | -0.090 |
| Resting Diastolic BP (mmHg) | 113 | 68.386 | 8.423 |  | 84 | 72.941 | 9.882 |  | -3.484 | <.001 |  | -0.502 | -0.788 | -0.215 |
| Resting HR (BPM) | 113 | 63.915 | 9.892 |  | 84 | 66.790 | 10.850 |  | -1.936 | 0.054 |  | -0.279 | -0.562 | 0.005 |
| RMSSD (ms) | 104 | 49.644 | 31.820 |  | 75 | 52.237 | 38.869 |  | 0.058 | 0.954 |  | 0.009 | -0.288 | 0.306 |
| Cardiometabolic Load Factor Score (z score) | 102 | -0.282 | 0.868 |  | 72 | 0.241 | 1.107 |  | -3.347 | 0.001 |  | -0.537 | -0.843 | -0.229 |
| Discrimination (EDS; 1-6) | 114 | 1.663 | 0.683 |  | 85 | 2.017 | 0.819 |  | -3.235 | 0.001 |  | -0.476 | -0.760 | -0.191 |
| Stress (PSS; 0-40) | 114 | 12.737 | 5.464 |  | 85 | 14.600 | 6.120 |  | -2.260 | 0.025 |  | -0.324 | -0.606 | -0.041 |
| Distress (SCL-90 GSI; 0-4) | 114 | 0.306 | 0.324 |  | 85 | 0.427 | 0.435 |  | -2.483 | 0.014 |  | -0.356 | -0.638 | -0.072 |
| Stress Factor Score (z score) | 114 | -0.195 | 0.910 |  | 85 | 0.164 | 1.043 |  | -2.582 | 0.011 |  | -0.370 | -0.653 | -0.086 |
| Pain Inhibition (CPM-NFR; change score) | 102 | -0.073 | 0.394 |  | 72 | 0.019 | 0.422 |  | -1.479 | 0.141 |  | -0.228 | -0.530 | 0.075 |
| Pain Amplification (TS-pain; change score) | 98 | 10.616 | 14.659 |  | 70 | 12.530 | 14.670 |  | -0.834 | 0.406 |  | -0.130 | -0.437 | 0.177 |

Note. BMI=body mass index. BP=diastolic blood pressure. HR=resting heart rate, beats per minute. RMSSD=root mean square of successive differences (measure of resting heart rate variability that assesses parasympathetic function). EDS=Everyday Discrimination Scale. PSS=Perceived Stress Scale. GSI=Global Severity Index of the SCL-90. CPM=conditioned pain modulation. NFR=nociceptive flexion reflex (a measure of spinal nociceptive processing). TS=temporal summation. Pain inhibition=more negative values indicate greater inhibition, and positive scores indicate impaired inhibition (facilitation). Pain amplification=higher positive scores indicate greater pain summation/amplification.
